# What Makes People With Chronic Illnesses Discontinue Treatment? A Practice Theory Informed Analysis of Adherence to Treatment among Patients With Drug-Resistant Tuberculosis in Pakistan

**DOI:** 10.34172/ijhpm.2022.6576

**Published:** 2023-01-28

**Authors:** Shazra Abbas, Michelle Kermode, Mohammad Dost Khan, Justin Denholm, Hamed Adetunji, Sumit Kane

**Affiliations:** ^1^Nossal Institute for Global Health, Melbourne School of Population and Global Health, The University of Melbourne, Melbourne, VIC, Australia.; ^2^Department of Infectious Diseases, University of Melbourne, Melbourne, VIC, Australia.; ^3^Faculty of Public Health & Health Informatics, Umm Al Qura University, Makkah Almukarramah, Saudi Arabia.

**Keywords:** Health Systems, Patient-Provider Relationship, Respectful Healthcare, Pakistan, Health Systems Responsiveness, Practice Theory

## Abstract

**Background:** Non-adherence to treatment is a frequently observed phenomenon amongst those on long-term treatment for chronic illnesses. This qualitative study draws upon the tenets of ‘practice theory’ to reveal what shapes patients’ ability to adhere to the demanding treatment for drug-resistant tuberculosis (DR-TB) at three treatment sites in Khyber-Pakhtunkhwa (KP) province of Pakistan.

**Methods:** This qualitative study involved observation of service provision over a period of nine months of stay at, and embedment within the three treatment sites and in-depth interviews with 13 service providers and 22 patients who became non-adherent to their treatment.

**Results:** Consistent with the extensive research based on the barriers and facilitator approach, both patients, and providers in our study also talked of patients’ doubts about diagnosis and treatment efficacy, side-effects of drugs, economic constraints, unreliable disbursements of monetary incentive, attitude of providers and co-morbidities as reasons for non-adherence to treatment. Applying a practice theory perspective yielded more contextualised insights; inadequate help with patients’ physical complaints, unempathetic responses to their queries, and failure to provide essential information, created conditions which hindered the establishment and maintenance of the ‘practice’ of adhering to treatment. These supply-side gaps created confusion, bred resentment, and exacerbated pre-existing distrust of public health services among patients, and ultimately drove them to disengage with the TB services and stop their treatment.

**Conclusion:** We argue that the lack of supply-side ‘responsiveness’ to patient needs beyond the provision of a few material inputs is what is lacking in the existing DR-TB program in Pakistan. We conclude that unless Pakistan’s TB program explicitly engages with these supply side, system level gaps, patients will continue to struggle to adhere to their treatments and the TB program will continue to lose patients. Conceptually, we make a case for reimagining the act of adherence (or not) to long-term treatment as a ‘Practice.’

## Background

**Key Messages**

** Implications for policy makers**

- Enabling adherence to long term treatments for chronic illnesses remains a challenge for health systems globally. Understanding and approaching adherence as a ‘practice’ can help overcome the challenge.
- Enabling patients to adhere to demanding and long-term treatments require health services to be not just available and accessible, but importantly, to be responsive.
- A health system that is not responsive to patients’ legitimate expectations loses their trust, ultimately leading to discontinuation of treatment.
- Responsive health services that foster active participation of patients in their treatment processes can enable patients to adhere to demanding, long-term treatment regimes.
- Practice theory provides a comprehensive framework to examine practices (of both patients and the providers) in a health system and their impact on health outcomes.

** Implications for the public**

Non-adherence to treatment can have serious implications for health. Non-adherence and the associated loss to follow-up among drug-resistant tuberculosis (DR-TB) patients is a major public health issue with detrimental effects not only for patients but also for their families and the community at large. This research highlights several hitherto insufficiently recognised supply-side gaps that negatively influence patients’ ability to adhere to their treatment despite their desire to do so. In addition, to providing free of cost healthcare, it is also the responsibility of the health system to make these healthcare encounters a respectful and pleasant experience for patients. A responsive health system, where patients are listened to empathetically, their doubts are clarified, and where they are actively involved in processes concerning their health can help improve their adherence to treatment.

Adherence to treatment is defined as “the extent to which a person’s behaviour – taking medication, following a diet, and/or executing lifestyle changes – corresponds with agreed recommendations from a healthcare provider.”^1^ It is estimated that more than 50% of people with a chronic illness, especially in the low- and middle-income countries do not follow their healthcare provider’s (HCP’s) advice and become non-adherent to treatment at some stage in their therapeutic journey.^2,3^

 Factors that shape patients’ adherence to treatment have been subject to scientific enquiry from a range of disciplinary and theoretical perspectives.^4^ The biomedical model casts HCPs in a paternalistic role and positions patients as passive recipients of ‘expert’ advice.^5^ Over the last few decades, a range of psychological theories such as the Health Belief Model, Social-Cognitive Theory and the Theory of Reasoned Action have critiqued the passive role of patients’ in healthcare and posited adherence as a function of an individual being informed, motivated, and convinced of its benefits and not just a passive recipient of expert advice.^6-9^

 However, despite having received up-to-date information, and maybe even motivated and convinced about the prescribed therapies, human-beings can act contrary to what is logically expected.^10^ These arguments are based on studies that investigated how our behavior and actions are shaped by our environments, social positions, and circumstances, and by the power relationships we are embedded in – most of which we have little agentic control over.^7,10^ Analytically, such an understanding highlights how social determinants and structural factors shape people’s actions and health-related behaviors.

 While these theoretical advances have broadened our understanding of the phenomenon of adherence to treatment, they have had limited influence on related public health policies and programs.^9,11^ A key bottleneck to achieving better adherence to evidence-based treatments has been the continued privileging of the biomedical paradigm and an emphasis on either individual and/or environmental influences. There is therefore a pressing need for a revised and more realistic-approach to investigation of (non)-adherence to treatment.^9,12^

 A practice theory informed analytical approach is one such promising alternative.^9,13,14^ Practice Theory emerged in the mid-twentieth century in the works of social theorists such as Bourdieu, Giddens, and Foucault and has since generated a range of perspectives.^9,13-16^ Despite notable differences in the various conceptualisations of practice theory, the common thread is the centrality of ‘practice’ as the unit of enquiry.^13^ Recently, Shove et al applied practice theory to public health by positioning ‘practice’ as the starting point if we want to understand what it takes for people (referred to as ‘practitioners’) to embrace a practice (such as taking daily medication) or abandon it.^9,13^ Practice theorists conceptualise ‘practices’ as “routinised types of human activity that are made up of and can be recognised by the coming together of their constituent elements.” These elements are usefully condensed into three by Shove et al.^13^

- Materialities: refers to ‘things’ or material inputs required to initiate and sustain a practice,
- Competencies: the knowledge and skills needed to perform a practice,
- Meanings: the symbolic significance of a practice for the practitioner.

These elements, through their content, arrangement, and interactions, enable a practice to happen, to be maintained, or to end.^9,17^ It follows that if any of the three elements or their content are compromised, the way a practice is performed by its practitioners may well change.^9^

 These practice theory concepts have been used to examine behaviours (treated as practices) such as smoking, snacking, and drinking alcohol, to understand why, despite investment at both individual and structural levels, these practices persist.^9,13,16,18^ Skovdal et alrecent work demonstrates the analytical potential of practice theory to understand patients’ (non)-adherence to and (dis)engagement with HIV services.^12,19^

 In this article we examine adherence to treatment as a ‘practice’ by applying the concepts of the three constituent elements (materialities, competencies, and meanings) to enrich and nuance the existing knowledge and understanding of adherence to treatment by asking two research questions; (1) What factors influence drug-resistant tuberculosis (DR-TB) patients to discontinue treatment? (2) What is the analytical potential of practice theory to strengthen our understanding of the factors contributing to DR-TB patients’ decisions to discontinue treatment?

###  Study Context 

 Pakistan is one of 20 countries in the world with the highest incidence of DR-TB.^20,21^ In response, the National TB Program (NTP) in Pakistan introduced a Programmatic Management of Drug-resistant TB (PMDT) model of care in 2010, which provides free healthcare to DR-TB patients.^22,23^ The PMDT model involved: establishment of exclusive PMDT clinics in selected tertiary hospitals across the country; appointment of a core health team including a physician, a pharmacist, a psychologist, and a treatment coordinator (outreach worker) in addition to four ancillary staff members encompassing a case manager (maintains patients files), a laboratory assistant, a data operator (maintains computerized clinic statistics), and a social support officer (manages financial support package to patients); free of cost DR-TB medicines and laboratory services; appropriate clinic opening hours (from 0800-1400 hours, 6 days a week); and a financial support package to cover patients’ travel costs.

**Figure 1:**
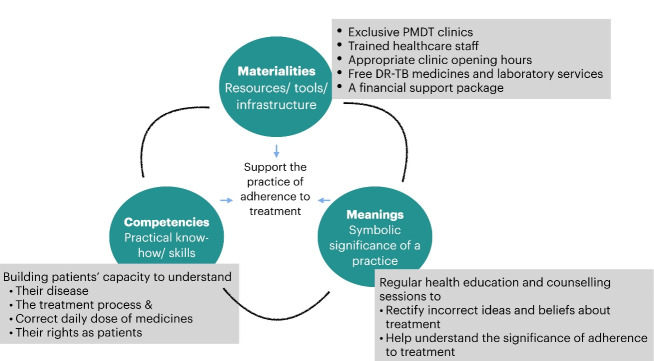
NTP’s Provisions for Materialities, Competencies and Meaning to Promote Patients’ Adherence to Treatment. Abbreviations: NTP, National TB Program; DR-TB, drug-resistant tuberculosis; PMDT, Programmatic Management of Drug-resistant TB.

 Beside these material inputs, through regular patient-provider communication, patients’ competencies are supposed to be built to help them understand their disease, the treatment process (treatment for DR-TB is expensive, long, and associated with more frequent and serious side-effects compared to the treatment for primary TB^22^), the correct daily dose, the side-effects of the medication, as well as the minimum standards of care they are entitled to receive at a PMDT clinic. Furthermore, regular health education and counselling sessions at PMDT clinic are set-up to address any misconceptions (the meaning-making by patients and their families) – both *a priori *and during treatment covering the nature of the disease, its curability, and the value of adherence to treatment (Figure 1).

 From a practice theory perspective, the availability and alignment of the three elements (ie, materialities, competencies, and meanings) helps the practitioners (patients) to embrace and sustain the practice of adherence to treatment and should lead to better health outcomes. Nonetheless, despite these inputs, the treatment success rate among DR-TB patients in Pakistan remains as low as 65%; with consistently high non-adherence and lost to follow-up (LTFU) being the main drivers.^21,24,25^

## Methods

###  Study Setting and Participants 

 This study was conducted in three PMDT sites in Khyber-Pakhtunkhwa (KP) province of Pakistan. The population of interest was DR-TB patients aged ≥18 years who had commenced treatment at a PMDT clinic, but subsequently became non-adherent for at-least two consecutive months which is the NTP definition of non-adherence and LTFU.^26^ The two terms (non-adherence and LTFU) are used interchangeably by the NTP. The core health team (physician, pharmacist, psychologist, and treatment coordinator) at each study site were interviewed. At one site the case manager was additionally interviewed due to his direct involvement in patient care. While this study does draw on staff interview data regarding their perspectives on patients’ (non)-adherence to treatment, the perspectives of the LTFU patients themselves are the primary focus of this paper. A detailed analysis of the providers’ interviews is published elsewhere.^27^

###  Data Collection Tools and Approach 

 Before commencing fieldwork, we had meetings with the NTP to understand the structure and functioning of the PMDT model of care. We reviewed literature on factors influencing patients’ adherence to treatment,^28-31^ and the NTP annual reports between 2010-2019.^21,25,32-34^ This preliminary work contributed to the development of guides for participant observation and semi-structured interviews. The topic guide for semi-structured interviews broadly encompassed two themes: understanding LTFU patients’ journeys from the time they first realized their disease symptoms and how they arrived at the PMDT clinic; followed by an in-depth exploration of these patients’ experiences in the PMDT clinic and what shaped their decision to ultimately cease treatment. The participant observation guide covered two broad thematic areas: the physical description of the PMDT clinic and daily workflow; and secondly patients’ interactions with core health team members and ancillary staff. The study tools were pre-tested at a PMDT clinic not included in the study, adjusted for minor revisions, and approved by the NTP.

 Data were collected between June 2018 and May 2019. The first author (SA) presented the study objectives and methodology to the PMDT teams, responded to their queries, and started fieldwork with their consent. SA lived within the hospital premises for 3 months at each study site and spent about 5 hours per day, 6 days a week, in the PMDT clinics. This arrangement allowed for extensive observation of the workflow and provision of healthcare services at each site. With prior verbal consent of patients and providers, SA also attended patient consultations to gain first-hand understanding of how adherence to treatment was promoted.

###  Recruitment of Study Participants

 At each study site, a list of all LTFU patients was provided by the clinic team with the approval of the NTP. Patients were contacted by the treatment coordinator by phone. The treatment coordinator introduced SA and the purpose of the study to them, and SA requested a one-on-one interview. If the patient agreed to meet, a home visit was scheduled. Each interview lasted for 40-60 minutes but typically involved spending about 2-4 hours at each patient’s home, meeting their families, and having informal discussions with them to better understand the context in which the decision to cease treatment was made. All interviews were digitally audio-recorded with participant consent. Four patients who had history of LTFU during the current course of treatment were interviewed in a private space within the hospital. No PMDT staff was present at the time of these interviews. Interviews with HCPs were conducted at a mutually agreed time and place; only data pertaining to their perspectives on patients’ non-adherence are drawn upon for this paper.

 SA’s background as a Pakistani national and a medical doctor with work experience in KP, her familiarity of local culture, and fluency in local languages enabled us to better apprehend patient accounts. The interview data was triangulated using approaches such as prolonged presence of SA in the field, recording extensive fieldnotes on daily basis, visiting patient homes using the same route and public transport as patients, and an informed comparison of what was being said vs. done in healthcare provision in the PMDT clinics. The preliminary findings were presented to the national and provincial TB programs at the end of the fieldwork.

 At the time of this study there were a total of 44 patients at the three study sites classified as LTFU. Of these, we were able to access 22. Seven declined to meet, and 15 could not be reached because they had died, were in prison, or were living in war-affected zones (Table 1).

**Table 1:** Lost to Follow-up Patients at the Study Sites

**Site**	**Total LTFU Patients**	**Interviewed **	**Refused **	**Died**	**Not Accessible**
Study site 1	14	6	6	2	0
Study site 2	7	6	0	0	1
Study site 3	23	10	1	7	5
Total	44	22	7	9	6

Abbreviation: LTFU, lost to follow-up.

###  Data Analysis

 The participant interviews were transcribed and translated verbatim^35^ by SA, who is fluent in both Urdu and English. QSR International NVivo 12 qualitative analysis software was used to manage data coding. We followed a step-by-step thematic analysis approach to arrive at our final themes.^36^ The early codes developed by SA were independently reviewed by another author (SK) to cross-check the adequacy and completeness of codes. Any disagreements were addressed through consensus. Ultimately, we agreed on seven themes that described reasons for patients’ non-adherence to treatment. The participant observation data was coded separately using the same approach. Finally, we interrogated and reinterpreted the themes in light of the three elements of practice theory to understand the often subtle nuances of non-adherence to treatment, which is our contribution to the existing knowledge on the subject.

## Results

###  A. Patients’ Demographic Data and Background Characteristics 

 The proportion of male patients was significantly higher than female patients. Almost all had a low level of education. Most of the men were breadwinners while all women were homemakers. Those who were working, mostly worked in menial jobs. About half of the patients had a family history of TB/DR-TB (Table 2).

**Table 2:** Patients’ Demographic Profile

**Variable**	
Gender	
Male	16
Female	6
Age (y)	
18-40	12
>40	10
Financial situation	
Breadwinners	9 (all men)
Earning independently with no family obligations	3 (all men)
Financially dependent	10 (4 men, 6 women)
Type of work	
Labourer	5
Skilled worker (tailor, mason, farmer, miner)	4
Faith healer	1
Domestic servant	1
Retired (on pension)	1
Unemployed	2
Student	2
Housework	6 (all women)
Education	
No formal education	13 (including all 6 women)
Primary or less	5
Secondly or high	4
Family history of TB/DR-TB	
Yes	10
No	12

Abbreviations: TB, tuberculosis; DR-TB, drug-resistant tuberculosis.

###  B. Factors Contributing to Patients’ Non-adherence to Treatment and Being LTFU

 This section responds to the first research question ie, what factors influence DR-TB patients to discontinue treatment? It primarily draws on patients’ accounts. Each primary reason shared by the patient at the time of their interview was probed further to deepen understanding of how it shaped their decision to cease treatment from the PMDT clinics.

####  Doubting or Rejecting the Diagnosis 

 A few patients were put on DR-TB treatment but were not convinced that they had the disease. For example, one patient queried discrepancies in his laboratory test results (done at two laboratories both operating under NTP) but never received a satisfactory explanation for the discrepancies from the PMDT staff. Instead, they simply advised him to take medication, which led him to doubt the diagnosis and ultimately, to stop treatment.

*P3: If a patient asks a question, staff should respond in a way that satisfies them. Staff should not respond like “this is the treatment, and you must take it” but explain with a reason. I am still confused [about my diagnosis], and I haven’t got any clear answer yet [from the clinic staff] as to why my test [for DR-TB] from one laboratory was positive, and from another laboratory it was negative*.

 A few younger patients rejected their diagnosis at the outset or after a few months of taking treatment. They felt that life had been unfair to them and that their families did not understand them. These patients rejected their diagnosis or refused to take the medicines to the extent that some reported inducing vomiting if the medicines were force-fed to them by family members. Among them, one was LTFU in the first month of treatment, while the other two had a history of LTFU more than once.

*P8: Yes, I had cough, but it was due to dust … but they (the PMDT staff) said I have this disease (DR-TB)…. They said I am weak (underweight) also, but I am not … my brother is also like me, but no one says that he has this disease. Actually, my family hates me…. I shouldn’t have agreed to go to this clinic (PMDT)*…

*P6:...In fact, I am not convinced that I have this disease … I left treatment and I am fine now*…

 HCPs, in their interviews, held families responsible for failing to help patients to accept their diagnosis. They also expressed the opinion that patients who ask a lot of questions were more likely to become LTFU. However, at least two HCPs identified weaknesses in the staff’s capacity to respond to patients’ queries as a factor driving them away from treatment.

####  Doubting the Efficacy and/or Legitimacy of the Treatment Course 

 There was another group of patients who accepted their diagnosis but still expressed their doubts about the efficacy and appropriateness of their treatment – these doubts, and the PMDT staff’s inability to address their concerns featured prominently in their explanation for stopping treatment. For instance, one patient attributed his recently diagnosed heart disease to DR-TB medication but did not receive a satisfactory explanation from the staff that the two health conditions were unrelated, so he discontinued treatment. Another patient, after having taken DR-TB treatment for three years, raised his concerns about the treatment efficacy. Instead of addressing his concerns, the clinic staff merely insisted that he continue treatment. He finally ceased treatment attributing his disease to supernatural causes.

*P18: He (staff) told me that my reports are not good. I said if the medicines are not helping me, then what is the purpose of coming to the clinic … I know this is sorcery. I am going to see a person who communicates with spirits/supernatural beings (called ‘Jinns’ in the local language). I have contacted him on WhatsApp. I am told that he can treat me*.

 Similarly, a few patients firmly believed that doctors (in general) over-treat patients and saw no reason to continue treatment once the disease symptoms had subsided.

*P11: I planned to take medicines for one year to get better instead of 2.5 years as they (PMDT staff) told me. I know that when doctors tell patients to take four tablets, patients should take two because, you know, doctors always over-prescribe*.

 While no HCP directly mentioned patients’ doubts about treatment efficacy as a cause of non-adherence, upon probing they reported that if patients do not see improvement in their health, they become anxious and may stop treatment. They also recognized that patients often had greater trust in the opinions of private practitioners and faith healers; and that this might drive discontinuation of treatment from the PMDT clinics and care seeking from other sources.

####  Side-Effects of Drugs 

 All patients in the study experienced at least some side-effects of DR-TB drugs including vomiting, joint pain, and diminished hearing. For some, the side-effects were severe enough to disrupt their daily routines. HCPs acknowledged debilitating side-effects as a reason for non-adherence to treatment. They explained that the cost of ancillary medicines (not provided for free by the NTP) to treat the side-effects and the physical and mental exhaustion wreaked by side-effects led some patients to discontinue treatment. However, patients’ accounts reveal a somewhat different reality. Patients reported visiting PMDT clinics to seek help to manage the side-effects, but they were rarely seen by the physician and usually attended by ancillary staff (who lacked the basic training and knowledge to manage the side-effects). These staff often dispensed DR-TB medicines despite side-effects, referred patients to a doctor outside of the PMDT clinic, or simply told patients to continue treatment. At times they dismissed patients’ concerns, often pushing responsibility back on to the patients or invoking destiny or God’s will.

*P15: My health deteriorated with the medication they gave me. I am not able to hear now, my vision also became poorer. I will not go back (to the PMDT clinic)*.

*I: Did you discuss these problems in the clinic*?

*P15: Many times … but they said they have already told us that this might happen. Hearing might or might not return, this is Allah’s will*.

 Unable to tolerate the side-effects, these patients sought help from other sources such as private practitioners who often prescribed primary TB medication, anxiolytics, anti-inflammatory medications, and steroids – all unsuitable treatments for DR-TB. Understandably, stopping the DR-TB medicines alleviated their side-effects convincing patients further that the treatment provided by the PMDT clinics was not right. At the time of their interviews, a few patients had completed the course of treatment prescribed by private practitioners and were convinced that they were now cured, while others were still taking treatment from private practitioners or faith healers. A small number had returned to the PMDT clinic after remaining non-adherent for months because private healthcare proved expensive. Some, after receiving unhelpful advice from the PMDT clinic staff, abandoned all hope of recovery and refused to seek care of any sort.

####  Economic Constraints Due to Inability to Work 

 For many patients, being on treatment meant being unable to work. They were manual laborers and needed to move to big cities in other provinces for better employment opportunities. However, to continue treatment for 18-24 months and to visit the PDMT clinic every month they had to stay in their home where there were often fewer work opportunities. Given their financial situation, these patients tended to remain adherent to treatment up until they felt they were healthy enough to return to work. They unilaterally decided that the purpose of taking the medications had been served so discontinued their treatment and returned to other provinces for work.

*P21: I improved with these (DR-TB) medicines. I started to walk again…. After 6-7 months I was all well. I continued medicines for 3-4 more months…. One day I thought that my health is better so why not stop the medicine for two days and see what happens*.* I did not take medicines for one week and felt even better, so I decided to (stop treatment and) return to Punjab (another province) for work*.

 The HCPs recognized this – noting that at times patients become so overwhelmed with their role as breadwinners that they don’t listen to advice and discontinue treatment as soon as they experience relief from symptoms.

####  Disruption in the Delivery of the Promised Payments 

 The NTP provides DR-TB patients with a monetary incentive to cover their travel costs to the PMDT clinic. For some patients receipt of this financial incentive was their sole motivation for attending the clinic. Consequently, when the NTP failed to provide the promised payments on a regular basis, many patients stopped attending the clinic. Irregularities in payments caused some to doubt the creditability of the broader program and this contributed to their decision to discontinue treatment.

*P4: We were told that 5000 rupees will be given to us every month, but we received only twice; once 500 rupees and then 600 rupees. Such things result in patients losing their faith in treatment. If the program fails to give the promised money, it might be that the medicines they give are also fake. Such things jeopardize the authenticity of the whole system*…

 For some patients, failure to provide the promised payment meant that they were unable to travel to the clinic.

*P7: In the last eight months I received nothing (from the clinic). I had no money to pay for travel. Neighbors avoid me, thinking I will ask for a loan…. Therefore, I stopped coming to the clinic*.

 HCPs recognized these concerns and agreed that gaps in the disbursement of incentives affected patients’ ability to travel. They also highlighted how the incentives sometimes diverted patients’ attention away from treatment to the financial benefits causing them to refuse treatment when there were disruptions in the payments.

####  Negative Attitude of Clinic Staff

 A few participants reported the disrespectful attitudes of some PMDT staff as their primary reason for stopping treatment. In one instance, a patient asked to be transferred to another PMDT clinic closer to his place of work. The clinic staff scolded him and warned him that he would not be welcome back. It so happened that the patient returned to the PMDT clinic area after a few months, and while he was keen on completing his treatment, he did not because of the staff’s response at that particular clinic.

*P10: As I returned (to my village), I did not go back to the (PMDT) clinic. I was told that if I start treatment from the other (PMDT) clinic then it would not be possible for me to return to this clinic. He (staff) shouted at me and said, “don’t come back to this clinic again.*”

*I: But was it that serious. I mean if you were back, you could have visited (the first PMDT) clinic to continue your treatment. *

*P10: No, no. He told me not to come back... that is why I did not*.

 While HCPs acknowledged that staff attitudes could have an impact on patients’ adherence to treatment, only one explicitly mentioned staff’s disrespectful attitude as a possible cause of non-adherence.

####  Experiencing Co-morbidities

 For some patients, concurrent health issues caused them to discontinue DR-TB treatment. In one instance, a patient suffered a stroke and was consequently in and out of care both in private and public hospitals. The patient’s family struggled to manage the two separate care processes, but ultimately became physically, emotionally, and financially exhausted to the extent that they abandoned all treatments, including DR-TB treatment.

*P13: She (patient) had paralysis. We took her to a hospital, where they (doctors) her to another clinic but then said we should take her home … she had continuous cough … we took her to another doctor, and he said she has brain hemorrhage, he gave some medicines and asked us to take her home. A doctor from the PMDT clinic then called us, but we were too tired of all the efforts … we refused to attend the PMDT clinic*.

 Co-morbidities as a cause of LTFU was noted by many HCPs. They explained that patients are sometimes mishandled in hospitals, which contribute to their discontinuation of all treatments including for DR-TB.

###  C. Application of Practice Theory-Strengthening Our Understanding of the Practice of (Non)-adherence to Treatment

 Section B describes seven factors as identified by the study participants which influenced patients to stop treatment. In Section C, we draw on participants’ accounts as well as our firsthand field observations of care provision at the three study sites and apply practice theory to reveal how the factors described in Section B are shaped by the gaps in, and misalignment of, the three elements of practice theory. This section responds to the second research question ie, what is the analytical potential of practice theory to strengthen our understanding of factors contributing to DR-TB patients’ decision to discontinue treatment? We demonstrate how materialities, competencies and meanings operate in tandem to enable or constrain a patient’s ability to adhere to treatment.

####  Materialities Matter to Support the Practice of Adherence 

 The PMDT model of care was designed (at least in theory) to fulfil a range of material inputs to support patients’ adherence to treatment (Figure 1). However, despite the best of intentions, the model as implemented in its real-world setting revealed the extent to which these materialities were compromised and their purpose sometimes lost.

 At all PMDT clinics, core staff responsible for patient care were the physician, the pharmacist, and the psychologist. However, in all three study sites, patients were rarely seen or clinically examined by the physician. The patients’ key contact at one study site was the psychologist while at another site it was the case manager who happened to have a background in nursing. At the third site, patients were seen discussing their health-related issues with *any* available staff member. Unsurprisingly, as also highlighted in Section B, given their lack of medical training, these ancillary staff were often not able to appreciate the clinical significance of patients’ complaints and sometimes gave advice that was insufficient or incorrect.

*P19: The doctor was rarely available in the clinic. No one in the clinic ever informed us (patients) that the doctor is available, and we can see him…. They only gave us a bundle of medicines*.

*I: Who gave you medicines*?

*P: The one with beard (lab attendant). *(This patient had nine clinic visits before he was LTFU).

*P8: She (patient) was not able to move her arm. Our whole family was disturbed because of that.... We took her to the (PMDT) clinic, but the doctor was not available. A staff examined her and asked us to go home and they would contact us... but no one contacted us.... After one month we took her to a private clinic.* (This patient was told by the private doctor that she did not have TB, and the medicines given at the PMDT clinic were fake). 

 Besides the physician, the pharmacist was another important staff member, whose role was to dispense medicines and counsel patients to manage side-effects of the medicines. However, pharmacists’ observed interactions with patients were also short and superficial. Medicines at two study sites were often dispensed by any available staff member with little or no discussion of side-effects or any other issues of concern to the patient or their family. On the few occasions when the pharmacist did consult with a patient, the interaction generally lasted for only a few minutes, and involved a one-sided, quick conversation with little attention paid to whether the patient understood what was being said.

 At two of the study sites, the psychologists were the most accessible staff members for patients. There was no psychologist at study site three during the time of data collection. Given the lack of direct involvement with patients on part of the doctors and pharmacists, the psychologists became the main resource for patients to discuss their treatment-related complaints. The psychologists dispensed medical advice despite having no training or mandate to do so.

*Observation Notes: The psychologist was talking to the patient. The physician entered the room. She told the physician that the patient complained of being sleepy all the time. The physician said, “that’s fine” and left the room. The patient murmured without addressing anyone “You cannot understand my problems.” The psychologist advised him to take four glasses each of water, “tang” (a sugary drink) and oral rehydration salts to stay alert. *(This newly diagnosed patient’s sister had died of DR-TB a few weeks earlier, after being LTFU for months).

 To enhance access to care, especially for those travelling from afar, all PMDT sites were supposed to remain open from 08:00-14:00 hours, 6 days a week. However, at all three sites, clinic staff determined their own work hours. Senior staff members invariably arrived late and left early, leaving the clinic in the hands of untrained ancillary staff. Occasionally, clinic staff covered for each other, leaving just 1-2 staff members to attend to what they deemed as the most important activities ie, receiving patients’ sputum samples, advising them of laboratory investigations, obtaining their signatures in a (financial incentive) logbook, and dispensing medicines.

*P5: No, I don’t have to wait in the (PMDT) clinic … it is in fact very quick. I come here, put my sputum sample in the (collection) box. The staff check my weight and ask me to sit in the waiting area. He then goes inside, brings me medicines, and tells me to go home. *(This patient had recent history of LTFU and returned only because he could not afford private care).

 This picture was consistent across the three sites – blood samples were regularly collected, chest X-rays ordered, and medicines promptly dispensed. However, the results of laboratory investigations and updates on disease progress were rarely discussed with patients, increasing frustration among many of them.

*P22: If we are only required to collect medicines and go back home, then how would we know about our disease progress. They should inform us about our test results. A person is on treatment for 8-9 months and gets no information about whether he is improving or not. Patients keep on taking medicines, which is so difficult. There are too many tablets…that badly affect the stomach. And patients have no idea if their health is improving or not. *(This patient left treatment prematurely based on his belief that he had recovered).

 To compensate patients travel costs the NTP provided them with financial support. However, none of the patients interviewed received this support reliably, making this a major source of dissatisfaction.

*P12: The amount that was given in the beginning was 3000-4000 rupees. Later it was reduced to 2200 rupees …We are not rich and most of us are laborers.… The money that they promised is not enough but even that small amount is not given regularly. *(This patient quit treatment due to prohibitive travel costs and severe side-effects).

 In summary, none of the material inputs, other than dispensing medicines and access to laboratory services, were provided as anticipated in the PMDT model of care.

####  Enabling Competencies to Sustain the Practice of Adherence 

 To examine patients’ knowledge of the disease and the treatment process as part of empowering them to sustain the practice of adherence we asked them a range of questions. We found that while participants were familiar with the term “TB,” very few understood what having “drug-resistant TB” meant or how their disease differed from “ordinary” (primary) TB. Most were not aware of any mode of TB/DR-TB transmission and several attributed it to fate, stress, and sharing food.

*I: Do you know how this disease spreads*?

*P2: I think it is due to worries. Doctor said that it is not necessary that the germs are always in sputum.... If so everyone in the world would have died.* (This patient had eight visits to the PMDT clinic before he was LTFU).

 To explore participants’ understanding of their entitlements as patients, we asked them about their routine when attending the PMDT clinic. Participants in general had limited understanding of the responsibilities of individual PMDT staff involved in their treatment. Despite remaining in treatment for months, majority were unable to correctly identify or name staff members according to their role in healthcare provision. Patients assumed that all staff were “*chotay*” (junior) doctors, while the physician was considered *“bara” *(senior) doctor who, given their *‘bara’ *status, was not always accessible. For most, the primary purpose of visiting the clinic was to submit their sputum sample and to collect medicines. No formal health education sessions were conducted for patients and their families at any of the three study sites. Nor were there any systematic pathways to promote patients’ understanding of their disease, treatment, or progress. The knowledge imparted to patients was often random, fragmented and at times incorrect, sometimes causing more harm than good.

*P14: I was at the (PMDT) clinic when I started sweating…. I requested him (lab assistant) to check my blood pressure. He said this (sweating) is due to these (DR-TB) medicines, but I shall be fine soon.... I came home and stopped taking these medicines. Then this person (treatment coordinator) visited me. He suggested I should try taking half the dose. I took half dose for 15 days but then stopped due to heartburn…. I was mentally disturbed … so I quit treatment and decided to go to a ‘peer’ (term used for a faith healer in the local language). Someone has put a spell on me*.

 None of the study sites had any protocols regarding information to be provided to patients and by whom; how much information to be imparted during initial and follow up visits. There were no attempts to create a two-way dialogue with patients and no processes put in place to assess a patient’s ability to absorb and enact what they were being advised to do. Except for a few, the clinic staff themselves lacked the competencies required for a meaningful and empathetic discussions with patients, let alone being able to build patient’s competencies.

####  Altered Meaning Making and its Impact on Continuing the Practice of Adherence 

 In this sub-section we demonstrate how materiality and competency gaps can erode certain *a priori* meanings and thereby contribute to the emergence of meanings that undermine the practice of adherence to treatment.

 When diagnosed with DR-TB, most patient participants promptly contacted a PMDT clinic, even though many lacked a clear understanding of the purpose of their referral, and already had long histories of healthcare seeking. Nonetheless, the fact that they promptly contacted a PMDT clinic as advised suggests that, at least initially, they believed their disease might be cured with the treatment available from the PMDT clinic. However, the care experiences of some patients undermined their initial commitment to treatment and contributed to their losing faith of ever being cured. Others ended up believing that they were misdiagnosed and given incorrect treatment or fake medicines.

 The patients’ lack of competencies to deal with the long duration of treatment, and the lack of support in coping with the side-effects of medicines eroded their optimism and hope-based meanings that informed their initial belief in the value of treatment. Similarly, a lack of detectable improvement in their health and inadequate explanations from staff paved the way for alternative meanings/explanations for their suffering (eg, possession by spirits, destiny) to emerge. The vacuum thus created by these gaps in materialities (from the program side) and competencies (from the patients’ side) was filled in by opportunistic private practitioners who tended to entrench mistrust of public services.

*Observation Notes: We (SA and the treatment coordinator) entered the patient’s home. She saw the treatment coordinator and started shouting. “Oh, you have come again to make me sicker. Your medicines weakened my arm. I still can’t move my arm. Don’t you dare ask me to come back to your clinic. My doctor (private) has told me that I have no TB.*”

 A few patients had no intention of completing their treatment course from the very beginning as demonstrated in section B. They understood being healthy as being well enough to return to work. Given the gaps in materialities and competencies, this meaning was never challenged, and the PMDT staff did nothing to address this misunderstanding. These patients left treatment once they had achieved their self-defined goal. Similarly, rooted in entrenched cultural notions often fueled by influential traditional healers, some firmly believed that TB medicines were not effective if not complemented with good diet and complete rest. Thus, many chose not to continue treatment because complete rest and good food (variously understood) were not possible for them.

*P20: Hakeem (traditional healer) told me that TB patients should not do physical exertion and should eat well. I was taking (DR-TB) treatment, but I had no money to buy good food. You know medicines alone cannot help if the diet is not good … so I stopped treatment*.

## Discussion and Conclusion

 The available literature highlights a range of factors that undermine patients’ ability to adhere to treatment for chronic illnesses and can be broadly grouped into: (a) patient-related factors (eg, poor knowledge about the disease, cultural beliefs)^29,37,38^; (b) socio-economic factors (eg, financial constraints, treatment costs)^30,38,39^; (c) regimen complexity (eg, long treatment duration, side-effects of medicines, pill burden)^31,37,39,40^; and (d) health system factors (eg, lack of infrastructure, uncaring attitude of HCPs, poorly trained/overworked HCPs).^31,39,41^

 The PMDT model of care in Pakistan was a legitimate response to many of these long-known barriers to adherence and was expected to enhance patients’ ability to adherence to treatment and reduce the proportion of LTFU patients. However, as documented in the NTP reports this has not worked as per plan.^21,24,25,42^ The limited number of studies published on the PMDT model of care in Pakistan also remain restricted to reporting on drug resistance patterns, treatment success and mortality rates.^43-47^ While these studies highlighted non-adherence to treatment as a contributor to poor patient outcomes none attempted to explore the causes of non-adherence especially not from the patients’ perspective. However, in at least one local study the authors explicitly queried why such a devoted healthcare model was not improving patients’ adherence to treatment.^47^

**Figure 2:**
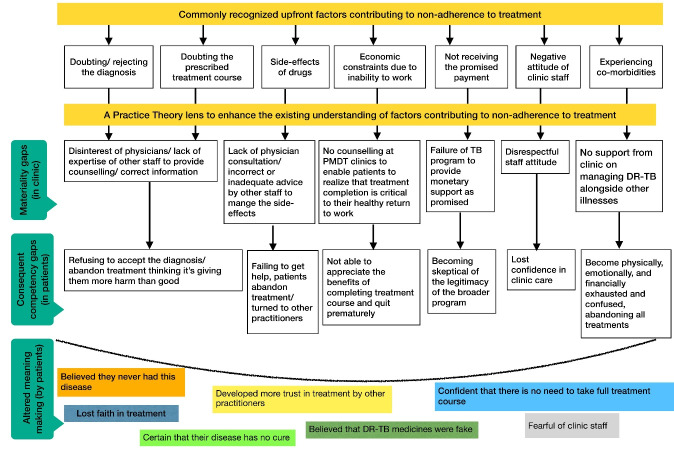
Application of Practice Theory to Further Interrogate the Upfront Factors Contributing to Patients’ Non-adherence to Treatment. Abbreviations: TB, tuberculosis; DR-TB, drug-resistant tuberculosis; PMDT, Programmatic Management of Drug-resistant TB.

 The upfront factors we presented in Section B engage with this knowledge gap and are consistent with findings from recent studies conducted at dedicated DR-TB centers in India, a context similar to Pakistan.^48,49^ However, in our analysis we went a step further and applied practice theory to methodically think through not only the upfront on-surface barriers to adherence but also the more subtle but decisive factors causing patients to discontinue treatment (Figure 2).

 Examining adherence as a ‘practice’ allows us to see how, paradoxically, the PMDT model as interpreted and implemented on-ground contributed to patients’ compromised capacity to adhere to treatment. Materiality gaps included disinterest of core technical staff in patient care, capacity gaps and disrespectful attitudes of ancillary staff, provision of inadequate/incorrect information to patients, and failure of the NTP to provide the promised payments. All these factors contributed towards patients’ limited competency to understand their diagnosis and adhere to the appropriate course of treatment. Additionally, they lost confidence in the healthcare staff and system, and in their own ability to recover from this significant chronic illness.

 These materiality and competency gaps compromised the effectiveness of this comprehensive model of care by reducing it to the performance of a few basic functions; namely collecting patient sputum samples, referral for a predetermined list of investigations, and dispensing DR-TB medicines. Critical functions such as listening to patients, responding to their queries, building their capacities and trust, and establishing linkages with other hospital services were either marginalized or delegated to ancillary staff who lacked the knowledge, authority and skills required to fulfil these roles.

 These materiality and competency gaps shaped, in a range of ways, the various meanings patients arrived at, regarding their illness and treatment, which subsequently influenced their decision to cease treatment. Some were (incorrectly) convinced that they were cured while others believed they never had TB in first place. They lost faith in the prescribed treatment and the trust they once attached to care provision at PMDT clinic, making them more inclined to seek care from other providers, and ultimately discontinuing treatment from the PMDT clinic. Each of these altered meanings was clearly the result of missed opportunities during the care encounter. A more empathetic and responsive patient-provider interaction, and a more accountable care process and health system^50^ would have fostered the maintenance and inculcation of more positive meanings, enabling patients to adhere to treatment and leading to much better outcomes for their health and wellbeing.

 Our analysis illustrates the analytical potential of practice theory to strengthen our understanding of the phenomenon of (non)-adherence to treatment. These findings can be used to improve the way public health initiatives are designed and implemented in real world settings. We conclude that, unless the NTP explicitly engages with these materiality and competency gaps and the resultant altered meanings, patients attending PMDT clinics will continue to struggle to adhere to treatment and the TB program will continue to lose patients. Finally, from a broader public health perspective, the failure to address these system gaps contributes directly to the erosion of patients and their families’ lifelong trust in not only PMDT clinical services but also other public health services, which has obvious long-term implications for individual and population health outcomes and the success of other similar initiatives in the country.

 This study has several limitations. Despite our best efforts we could only reach half of the LTFU patients from the three participating clinics. The inclusion of these missing patients, especially those who refused to be interviewed, might have revealed additional factors contributing to non-adherence. Secondly, the fact that contact with patients was mediated via the PDMT staff may have inhibited what they were willing to reveal to the researcher, at least to some extent. The impact of these limitations was minimized by the researcher’s reassurance to participants that she was not part of the clinic team, guaranteeing anonymity, and ensuring that interviews were conducted in private.

## Ethical issues

 This study was approved by the ethics committees at the University of Melbourne, Australia and the Health Services Academy in Islamabad, Pakistan. A plain language statement in English and Urdu (the local language) was provided and informed consent taken from the participants. Verbal consent was recorded and saved for patient participants not able to read and write. Three patient participants preferred a family member to speak on their behalf; in these cases, the interview was conducted with the assigned family member alongside the patient. Administrative approval for the study was obtained from the national and provincial (KP) TB programs in Pakistan.

## Competing interests

 Authors declare that they have no competing interests.

## Authors’ contributions

 Conception and design: SA. Acquisition of data: SA. Analysis and interpretation of data: SA, MK, JD, MDK, and SK. Drafting of the manuscript: SA, SA, HA, and SK. Critical revision of the manuscript for important intellectual content: SA, SA, SK, and HA. Administrative, technical, or material support: SK. Supervision: SK and SA.
